# Extracellular DNA (eDNA): Neglected and Potential Sources of Antibiotic Resistant Genes (ARGs) in the Aquatic Environments

**DOI:** 10.3390/pathogens9110874

**Published:** 2020-10-23

**Authors:** Periyasamy Sivalingam, John Poté, Kandasamy Prabakar

**Affiliations:** 1 Department F.-A. Forel for Environmental and Aquatic Sciences and Institute of Environmental Sciences, School of Earth and Environmental Sciences, Faculty of Science, University of Geneva, Uni Carl Vogt, 66 Boulevard Carl-Vogt, CH-1211 Geneva 4, Switzerland; john.pote@unige.ch; 2 Postgraduate and Research Department of Microbiology, Jamal Mohamed College, Tiruchirappalli 620020, Tamil Nadu, India; 3 Postgraduate and Research Department of Zoology, Jamal Mohamed College, Tiruchirappalli 620020, Tamil Nadu, India; drpklab@gmail.com

**Keywords:** aquatic environments, extracellular DNA, eDNA isolation, eARGs, environmental antibiotic resistome

## Abstract

Over the past decades, the rising antibiotic resistance bacteria (ARB) are continuing to emerge as a global threat due to potential public health risk. Rapidly evolving antibiotic resistance and its persistence in the environment, have underpinned the need for more studies to identify the possible sources and limit the spread. In this context, not commonly studied and a neglected genetic material called extracellular DNA (eDNA) is gaining increased attention as it can be one of the significant drivers for transmission of extracellular ARGS (eARGs) via horizontal gene transfer (HGT) to competent environmental bacteria and diverse sources of antibiotic-resistance genes (ARGs) in the environment. Consequently, this review highlights the studies that address the environmental occurrence of eDNA and encoding eARGs and its impact on the environmental resistome. In this review, we also brief the recent dedicated technological advancements that are accelerating extraction of eDNA and the efficiency of treatment technologies in reducing eDNA that focuses on environmental antibiotic resistance and potential ecological health risk.

## 1. Introduction

Risks of antibiotic-resistant bacteria (ARB) occur in both developed and developing countries. As a result, antibiotic-resistant bacteria are widely recognized as the biggest challenge in achieving universal health care, food security and development [1]. It has been reported that antimicrobial resistance associated infections kill more than 65,000 and 25,000 people in the United States and Europe per year, respectively [1,2]. The prudent and misuse of antibiotics in the health care sector, animal husbandry, poultry farming, along with agriculture, resulted in the release into the environment. This correlates with and likely contributes to antimicrobial resistance (AR) in human pathogens, rendering some infections untreatable. Antibiotic-resistance genes (ARGs) are now considered to be one of the emerging pollutants in the environment [3,4]. More seriously, antimicrobial resistance (AMR) has been estimated to cause 10 million deaths annually by 2050 [5]. The WHO recognized that AMR was one of the critical challenges of world public health in the 21st century [6]. Although the spread of antibiotic resistance is well-recognized in clinical settings, major limitations in understanding the environmental antibiotic resistome are one of the knowledge gaps to limit the spread. Therefore, this necessitates the implementation of the so-called one-health approach that brings humans, animals, and the environment together as one worldwide strategy to halt antibiotic resistance and its risk in the environment [7,8]. Waters are exposed to ARG pollution through diffuse (e.g., agricultural soils, extensive animal farming, urban and industrial areas) and punctual sources. Wastewater treatment plants (WWTPs), collecting sewage waters from urban areas, industries and hospitals, are considered the most prominent and globally diffuse hotspots for ARG released into the environment. This is caused by a general inefficiency of conventional WWTPs in abating ARGs, while co-selection mechanisms enhancing the spread of ARGs are promoted by treatments designed to reduce other pollutants (e.g., phosphorus and organic matter) from the sewage.

Recent studies enlightened the fate of bacteria carrying ARGs in WWTPs both during the treatment and in the final effluent, and large surveys allowed speculations on the possibility for such bacteria to survive within natural communities in water bodies receiving WWTP effluents. Furthermore, the fate of resistant bacteria and their ARGs when exposed to heavy metals, micropollutants, antibiotics, or when treated with innovative systems has lately been investigated [9,10]. This necessary body of research focuses on ARGs carried by intact (and generally active) bacterial cells, while it is well-known that the drastic treatments imposed to the sewage in WWTPs cause mortality of 90–99% of the bacterial community, resulting in a large amount of dead and broken bacterial cells and of free genetic material called extracellular or eDNA, is released into the environment within the WWTP effluents. However, the interest of the environmental microbiologist in eDNA in the aquatic ecosystems concerning its persistence and fate in the ARGs dissemination is very recent.

According to Corinaldesi et al, 2008, eDNA is defined as DNA, that is not associated with living biomass [11]. Recently, Torti et al., 2015, defined eDNA as that which is not enclosed in living cells [12]. eDNA has also been proposed as a ubiquitous biopolymer in the aquatic environment [13] and structural component of bacterial biofilms [14]. Besides, eDNA can act as a nutrient source for microorganisms in the environment [12,15,16]. Consequently, the occurrence of eDNA has been studied in various habitats, including soil, sediments, freshwater and marine ecosystems [17]. Both live and dead cells contribute to the release of eDNA as fraction to the environment in the different processes of secretion and lysis, respectively [18,19]. Given the long persistence of eDNA in sediment and soil samples for years, it is of utmost importance to study the role of eDNA in the spread of ARGs in the environmental matrices [18]. Recently, with the advancement of eDNA extraction technologies, many studies have reported that the occurrence of eDNA and ARGs in anthropogenic impacted environmental matrices including, surface water, soil, sediments, sludge, wastewater and tap water [18,19,20,21,22,23]. However, environments are as yet under-investigated as transmission routes and reservoirs of antibiotic resistance. Because eDNA could act as a potential genetic source of ARGs proliferation, in this review we will, therefore, summarize the studies that provide data on the quality of aquatic ecosystems (subject to WWTP effluent waters), in particular in predicting the role of eDNA in the context of environmental antibiotic resistome. Collectively, this review will be an improved understanding of the circulation of a key, but a neglected genetic material, in aquatic environmental compartments (water and sediment) using the advanced eDNA isolation technologies and bacteriological aspects based on molecular approaches.

## 2. eDNA Persistence and Natural Transformation

In general, the concentration of eDNA in sediments is known to be higher than in the water column in the marine environments [13]. The similar trend was also observed in freshwater environments. For instance, recently, Mao et al., 2014, demonstrated that a higher prevalence of ARGs in eDNA than iDNA collected from sediment and water [20]. When eDNA is released from cells, which may be adsorbed into soil, sediments, clay minerals and humic substances and likely to be prevented from degradation by extracellular nucleases [12,13,18,24]. On the other hand, eDNA that is not bound to the particle matrix can be present in the form of free–eDNA (f-eDNA) and can be degraded within several days [25]. Moreover, it has been reported that the efficiency of bacterial uptake to f-eDNA is easier than adsorbed eDNA [26]. For the physical persistence of eDNA in the environments, several mechanisms have been proposed. It has been reported that inorganic cations bridge to the negatively charged clay minerals, sand and phosphate groups of eDNA preventing its degradation and promoting persistence in the environments [12]. Low pH could also slow down the degradation of eDNA [17,27]. eDNA adsorption into the sediment matrix has also been reported to protect its degradation by nucleases [12]. Low temperatures are also found to be associated with decreased decay of eDNA in the environment [27]. Furthermore, oxygen availability and light have also been known to influence the persistence of eDNA in aquatic environments [28].

Indeed, ARGs have been reported to present in both intracellular DNA (iDNA) and extracellular DNA (eDNA) in the environment [4,20]. Antibiotic resistance genes (ARGs) from both iDNA and eDNA into the aquatic environment create hot-spots for horizontal gene transfer (HGT) and can accelerate the spread of antibiotics resistance [20,29]. The different mechanisms suggested for the transfer of intracellular DNA (iDNA) are conjugation or transduction. Whereas, the transformation process has been proposed as the only mechanism for the transfer of eDNA to naturally competent bacteria [30]. The first study, which emphasizes the capacity of bacteria to take up eDNA was published in 1928 [31]. With the spotlight on this study, Avery et al., 1944, confirmed that the transforming factor was really eDNA [32]. Notably, both linear and circular DNA has been reported to contribute to natural transformation in bacteria [30]. For evidence, the transformation of *Acinetobacter baylyi* strains isolated from soil to fragmented and damaged DNA was observed [33]. Natural transformation may vary in different bacterial species due to the expression of their competencies at different stages [34]. For instance, Ray and Nielsen, 2005, demonstrated that *Acinetobacter baylyi* attain maximum competence for natural transformation in the early and late exponential phases of the growth cycle [35]. It should be noted that structural proteins associated with eDNA are reported as not affecting DNA-binding or uptake [36]. Similarly, the eDNA that bound onto humic substances and proteins may not affect the transformation efficiency of the natural competent bacteria in soil and sediments. This hypothesis was proved in an experiment analyzing cell lysates which have also been shown to transform competent bacteria efficiently [37].

The natural transformation process could be one of the functionally important mechanisms in the environment due to many phylogenetically divergent bacteria, including Gram-positive such as cyanobacteria, Gram-negatives and archaea have the ability to acquire genetic traits of eDNA [38,39]. Interestingly, a research by Zhang et al. 2018 provides the first comprehensive insight into the role of eDNA in the dissemination of ARGs in an environmental microbial community by transformation [40]. Two other in situ researches confirmed the prominent contribution of eDNA in river sediments and biofilms [4,20]. Given the published papers, it has been understood that either purified chromosomal DNA or mobile genetic elements (MGEs) such as plasmids containing antibiotic resistance gene markers were shown to transform to natural competent bacteria in microcosm studies. These studies were conducted in ground water, river water, sediments, soil and marine water and sediments [37,41,42,43,44,45,46]. However, to the best of our knowledge, there were no studies on the extracted environmental eDNA for its transformation efficiency of the environmental bacteria to acquire antibiotic resistance. It has become apparent that extracellular ARGs (eARGs) present in eDNA acting as a dynamic gene pool could be attributed to the dissemination of antibiotic resistance through a natural transformation by competent environmental bacteria [8,20]. Furthermore, Yu et al., 2017 suggested that e-ARGs may also be the components of MGEs such as plasmids and integrons which can have the potential for rapid spread in bacterial population and contribute to the acquisition of antibiotic resistance by horizontal gene transfer [47]. In fact, several studies suggest that eARGs can persist in wastewater effluents even after several advanced treatment technologies, including: membrane filtration, chlorine disinfection and ultraviolet light disinfection. Consequently, the released eARGs into the aquatic environment may contribute to the sources of antibiotic resistance both in environmental and pathogenic bacteria [40,48,49,50].

## 3. Recent Advancements in eDNA Isolation from Water, Sludge and Sediment Samples

Isolation of pure eDNA with sufficient concentration is needed to understand how this genome remains stable in environmental matrices, and also to characterize how microbial communities respond to natural transformation among individuals, which we currently lack. Consequently, recent researches indicate that eDNA of surface waters and sediments contribute significantly to antibiotic resistome and impact risk for the environment. Fortunately, the isolation of eDNA from surface waters has dramatically increased in recent years. During the same time, thanks to the cost-effective next generation sequencing (NGS), have led to an increasingly sophisticated understanding of eDNA concerning environmental antibiotic resistome. Therefore, we highlight studies that demonstrate the isolation strategies to uncover how eDNA contributes to environmental antibiotic resistome and its impact on environmental risk. Generally, so far a very few efficient methods for isolating eDNA from water and sediment samples have been reported and can be classified into three major types, including surfactant cetyl trimethyl ammonium bromide (CTAB), nucleic acid adsorption particle (NAAP) and magnetic beads method and are summarized in Table 1. Furthermore, we believe that development of new methods will continue with further advancement of metagenomic approaches to extract eDNA.

### 3.1. Cetyl Trimethyl Ammonium Bromide (CTAB) based Method

The CTAB method was considerably effective, and many studies have followed in water and sediment samples [4,20,40,51,52]. In addition to surface water; sludge and sediment are considered to be sinks for pollutants including eARGs. Overall, this method was considered the most effective for the simultaneous extraction of eDNA and iDNA both from water and sediments. However, a recent study indicated that this method may not be suitable for the efficient recovery of f-eDNA in the aquatic samples where f-eDNA is predominant [17]. This method is based on the use of microfiltration (0.22 µm), alkaline phosphate buffers, enzyme (proteinase K) and different centrifugal forces. In particular, in sediments, eDNA that bound to clay minerals, humic substances, total organic carbon [20] and extracellular proteins should be extracted in higher concentrations and free from iDNA contamination [13,17]. The CTAB method involves a sequential extraction procedure in which eDNA can be isolated without causing cell lysis both in water and sediment. It has been demonstrated that alkaline phosphate buffer can desorb DNA that was adsorbed into the sediment matrix as there would be a competition between phosphate ions and the phosphate groups in the sugar-phosphate backbone of DNA [12,53]. The mild proteinase K treatment could remove extracellular proteins that bound on eDNA. The micro filtration using membrane could remove the contaminating bacterial cells and viral particles.

### 3.2. Nucleic Acid Adsorption Particles (NAAPs)

An increase in the discharge of treated domestic and agricultural wastewater directly into rivers and lakes and their tributaries has caused a significant decrease in water quality. The sediments, as well as the water column, were found to have significant sources of organic carbon, phosphorus and nitrogen. Recently Wang et al., 2020, reported that eutrophic water (high N and P content) was found to be suitable for the propagation of ARGs [54]. Therefore, it is important to have a desirable quantity and quality of eDNA concentration for quantitative real-time (qPCR), NGS and also bioinformatic analysis, which may influence the diversity and abundance of eARGs in environmental matrices. A recent study by Wang et al., 2016, developed a novel technology to extract eDNA from a large volume of water samples using an aluminum hydroxide column [55]. The higher specific surface area of NAAPs increases the adsorption of electronegative eARGs to electropositive NAAPs. The eluent that contains glysine at 0.05 mol/L showed high efficacy in the eARGs recovery. The hydrophilic nature of glycine can disrupt the hydrophobic interaction between NAPPs and eARGs and thereby creating a negative electrostatic interaction resulting in the dissociation of eARGs from NAAPs [55,56]. This technique has also deserved particular attention to isolate eDNA in oligotrophic water samples, including tap water, where the number of bacteria (and possibly also of eDNA) is very low [19]. The procedure described in this method was found to be useful for the extraction of both eDNA and iDNA from a large volume of water samples. However, the increasing efficiency in the recovery of eARGs from large volumes of water samples was observed in NAAPs; the authors have not been tested for sediment samples.

### 3.3. Magnetic Beads Method for eDNA Extraction 

Fascinatingly, a recent study demonstrated the use of magnetic beads in the isolation of eDNA of two different forms (adsorbed (a-eDNA) and free (f-eDNA)) from WWTP effluents and an activated sludge sample [57]. The authors have also optimized sample volume and magnetic bead loading. For the efficient recovery of eDNA using magnetic beads, the following mechanisms have been postulated. Similar to the NAAPs column, magnetic beads have also been reported to have high surface area which can provide more binding sites for DNA [58]. Cation bridging to the phosphate backbone of DNA and the hydroxyl group on the beads could provide high affinity for eDNA due to the stronger negative charge than other bound substances like proteins and humic acids [59,60]. This study has also identified the effectiveness of different pretreatments including the addition of Tris-EDTA (TE) buffer, phosphate buffer (pH = 4), shaking at 150 rpm for 20 min, 0.2 g polyvinylpyrrolidone (PVPP) and 20 mL phosphate buffer, then vortexing the sample, and just vortexing the sample before filtering through a 0.22 μm filter. According to Yuan et al., 2019b, magnetic beads were highly effective in the isolation of high concentration eDNA (A_260_/A_280_ value of 1.7) in a minimal volume of sample about 5 mL. Regarding pretreatments, vortex pretreatment for 20 min was found to be effective and yielded a high concentration of eDNA [57]. 

## 4. eDNA as Sources of Antibiotic Resistance in the Environment

**Figure 1:**
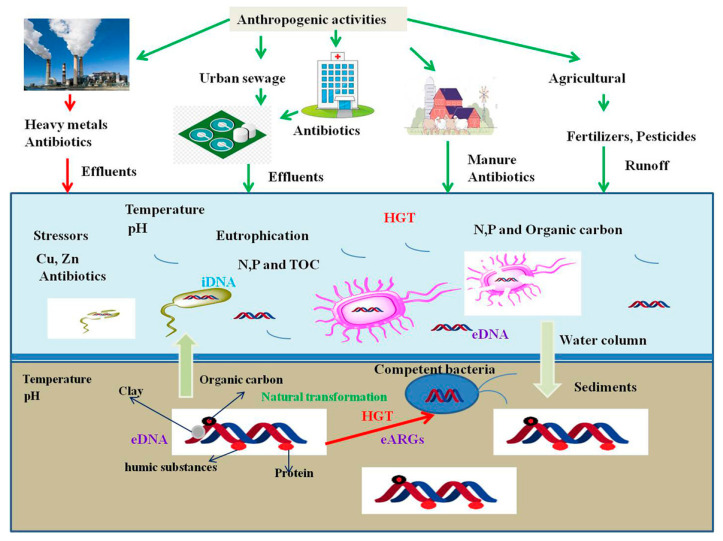
A schematic representation eDNA release and persistence and natural transformation in the aquatic environment under the influence of various biotic and abiotic stresses.

The spread of ARB and ARGs into the environment is a critical issue in combating antibiotic resistance worldwide. Recently, increasing numbers of studies suggest that eDNA act as potential sources of ARGs and contribute significantly to environmental antibiotic resistome. It should be noted that the environmental matrices which are highly influenced by anthropic activities, in particular urban sewages, animal wastewater, hospital wastewater, agriculture runoff and manure appliance are correlated to the high abundance of eARGs (Figure 1). The fate of ARB and ARGs released into the environment is related to a number of different external factors. When eDNA is released into the environment, that can interact with resident microbial communities, spreading by horizontal gene transfer (HGT) or by natural selection towards the allochthonous ARB. This can lead to the promotion of threatening reservoirs of resistances in environments exposed to highly inhabited areas or a direct transposition of ARB and ARGs. It has become problematic through irrigation of agricultural products or leisure activities, back to humans where they can promote the establishment of new antibiotic-resistant infections.

Surveillance is considered to be an important tool for managing antibiotic resistance [64]. ARGs encoded on eDNA belonging to different groups from investigated geographical regions are shown in Table 2. The distribution and abundance of eARGs showed a significant variation depending on the specific environment and sample types. The detection of various antibiotic resistance determinants in the investigated studies emphasized the importance of aquatic ecosystems as reservoirs of multiple eARGs. The eARGs most commonly detected were sulfonamide and extended spectrum beta-lactamase (ESBL) resistance determinants, and the least detected eARG was carbapenem resistance (Table 2). eARGs concern to carbapenem resistance was only reported from WWTP effluents (Portugal) and surface water from Coastal Bay (China). These findings suggest widespread dissemination of ESBL resistance determinants in the aquatic environments. Thus, in recent years, an increasing number of studies that support eDNA as present in environmental matrices and their global importance is associated with antibiotic resistome have been performed.

More recently, Oliveira et al. (2020) documented eARGs belonging to antibiotic class carbapenem (*bla*_KPC_, *bla*_OXA-48_, *bla*_NDM_, *bla*_IMP_ and *bla*_VIM_) and quinolones (*qnrA*, *qnrB* and *qnrS*) from WWTP influent, effluent and reused effluent samples [23]. The authors stated that of all the eARGs measured in the sample analyzed, *bla*_VIM_, *qnr*B and *qnr*S were detected in both the discharged and reused effluents with no significant changes in the concentration. These findings suggest that current conventional methods are ineffective in the complete removal eARGs from treated and reused effluents. This study suggested that the source of eDNA in the analyzed samples was probably derived from both cellular extrusion or cell lysis, an extracellular polymeric matrix of bacteria, humic substances and adsorbed on the colloids, sand particles and clay minerals [23]. Due to this, the authors raised concern over the reuse of WWTP effluents, and that it may have negative impacts on human health and the ecosystem and also recommended advanced treatment methods to remove eDNA.

Another recent study based on the metagenomic approach reported that eDNA retrieved from urban river water that encodes antibiotic resistance determinants included *sul1*, *tet(A)*, *ere(A)*, *bla*_TEM_ and *qnrD* [63]. eARGs was detected in all the downstream samples suggesting the impact of wastewater discharged into the river may reinforce the enrichment of eARGs [63]. The authors ascertained that adsorbed eDNA could play an important role in the spread and dissemination of ARGs in surface water. A similar finding was reported by Dong et al., 2019, who ascertained that adsorbed eARGs exhibited higher transformation efficiency than that of free eARGs [18]. In municipal sewage sludge, Lu et al., 2020, reported the presence of eARGs of different antibiotic classes such as aminoglycoside, tetracycline, chloramphenicol, sulphonamide, vancomycin, multi drug and beta-lactam in sludge samples [65]. The results indicated that the conditioning of sewage sludge is not effective in the removal of all eARGs analyzed. It was found that conditioned sludge accumulated eARGs, including *aadA-01, aadA-02, aadA1, aadA2-03* and *strB* and warrants future studies [65].

Likewise, the abundance of eARGs was reported in estuary sediments from China [61]. This study showed that estuary sediments are also one of the reservoirs of eARGs. Besides, the authors observed the high diversity and abundance of eARGs (*IntI-1(*clinic), *aad*A-01, *aad*A1, *aad*A2-03, *tet*G-01, *tet*X, *qacE*delta1-01 and *qac*H-01) which may be attributed to the high frequency of HGT to disseminate ARGs in the competent bacteria [61]. It was observed that the abundance of eARGs was relatively higher than that of iARGs in sediments. These results are consistent with previous studies which indicated that the abundance of eARGs in estuarine and coastal sediments is frequently high [4,62]. The authors also concluded that MGEs and bacterial communities are the major factors influencing the ARGs in the estuary sediments.

Dong et al., 2019, reported different eARGs (*sul1*, *sul2*, *tetW*, *tetX*, *ermA*, *ermB*, *bla*_TEM_, *ampC*, *cat* and *cmr*) and class I integron (intI1) in the sludge samples collected from hospitals, pharmaceutical industries, WWTP, swine manure and sediment in an urban lakes [18]. The results indicated that eARGs is usually lower than that of iARGs in sediments, and this finding was contrasted with the study, which was performed in estuary sediments, where the abundance of eARGs was higher [61]. Interestingly, the authors also found the highest ratio of eARGs in hospital sludge, followed by urban lake sediment, reflecting that hospital sludge could be an ideal pool for eARGs. Another key finding of this study was that adsorbed eDNA showed an increased transformation efficiency than that of free eARGs to competent *Escherichia coli E.coli*.

Interestingly, the distribution and relative abundance of eARGs in tap water samples sampled in Tianjin, China, were investigated by Hao et al., 2019 [19]. The results showed seasonal variation in eARGs abundance with the highest abundance in summer. *tetC* was found to be the most abundant eARG among all investigated ARGs. The authors proposed that the *tetC* gene might be considered as an eARG pollution indicator marker in tap water [19]. Yuan et al. (2019) investigated the occurrence of eARGs in sediments collected from two different aquaculture farms [21]. The *sul*1 and *tet*C genes were the most abundant eARGs among all measured ARGs. It was also observed that the abundance of ARGs is related to the types of ponds and rearing practices. This study also highlighted that antibiotic use was not correlated with the identified ARGs and that could be the result of co-selection stressors such as by the presence of metals and polyaromatic hydrocarbons [21].

Yuan and coworkers (2019b) investigated the abundance of eARGs in WWTP effluent water samples [57]. The report confirmed the higher prevalence of eARGs in the effluent. The results showed that activated sludge contains more ARGs than the influent. It was also observed that activated sludge harbored more ARGs than influent. The authors proposed that the death of host ARB in the activated sludge could be a significant source of eARGs. In the effluent, f-eARGs was found to be dominant, which contributes (90.3 ± 16.5%) of total ARGs. Although there was a significant reduction in the abundance of iARGs and a-eARGs in effluents, f-eDNA concentrations, had increased in the discharged effluents. This may result in the dissemination of ARGs into indigenous bacteria and pose a potential threat to human health and the environment [57].

The report by Sui et al. (2019) indicated that swine wastewater could be another potential reservoir of eARGs. In general, there has been a lower abundance of eARGs than that iARGs in untreated swine wastewater [51]. This study reported the most dominant resistant gene in eDNA in the discharged effluents was *sul*1. According to their findings, nitrate, organic matter and microbial community structures were found to positively correlate with the eARGs of discharged effluent.

Zhou et al., (2019) investigated the abundance and occurrence of eARGs in activated sludge waste from five wastewater treatment plants [50]. Although there was a major difference observed in the distribution pattern of species abundance between municipal WWTPs (n = 4) and swine WWTP (n = 1), all municipal WWTPs had shown no significant difference. The authors identified eARGs responsible for elfamycin, dual drug and aminoglycoside. Elfamycin and *intI1* were the most abundant genes that were encoded in eDNA. The authors also confirmed the co-location of eARGs and extracellular mobile genetic elements (eMGEs), including *sul-3’CS-TnAS3*, *sul2-intI1-ISVsa3* and *tetX-p63039*. These findings suggest the widespread of sulfonamide and tetracycline resistance genes in the environment which is consistent with the results presented by a previous study by Hao et al., (2019) [19]. The authors also proposed that abiotic selective stressors such as antibiotics and heavy metal might influence the relative abundance of eARGs in the environment and this finding was corroborated by a previous study [61]. In this study, the relative abundance of eARGs in activated sludge was found to be high. These results are in agreement with activated sludge which has been proposed as an important reservoir of eARGs [9]. In addition, the authors stated that nonspecific lysis of microbial cells could have contributed to the origin of eARGs.

The study by Guo et al., 2018, confirmed that estuary sediment impacted by anthropogenic activities serves as the potential source of eARGs [4]. The authors reported that *sul*1, *sul*2, *tet*A and *tet*W were the most abundant eARGs in biofilms and sediments. The results of this study have also demonstrated that the ARGs abundance in sediments is relatively higher in eDNA than in iDNA. Organic carbon, and metals (e.g., Zn and Cu) and antibiotics were identified to correlate with analyzed ARGs and indicating that selective pressure could attribute the selection of eARGs in sediments and biofilms [4].

In another study, the abundance and distribution of eARGs present in the surface sea water from Coastal Bay were conducted. The study confirmed that the presence of different antibiotic resistance determinants including *sul*2, *tet*B, *tet*M, *bla*_TEM_, *bla*_OXA-1_, *qnr*S and *oqx*B [62]. The authors reported that of the all eARGs investigated, *Sul*2 was the most abundant eARG in the water and sediment samples. In addition, the abundance of eARGs in the seawater was found to be higher than the levels of iARGs. In contrast, only *tet*M was detected in all water samples implying that seawater can also be an important reservoir of eARGs. This study also confirmed the influence of antibiotics, dissolved oxygen (DO) and pH on the occurrence of ARGs in seawater. The authors also emphasized the correlation found between *int*1 and *sul*2 from eDNA in this study, indicating horizontal gene transfer via natural transformation.

Effluents from WWTP after biological treatment, sludge settling, membrane filtration and disinfection was tested for the presence of eARGs. The authors found that treated wastewater could also contain significantly different antibiotic class resistance determinants for tetracyclines, sulfonamides, macrolides and β-lactams [40]. The results indicated the persistence of eARGs in the discharged effluents in all treatment processes (biological treatments, ultrafiltration, ozonation and chlorination) and had a lower decay rate than that of iARGs.

Interestingly, in another study, Liu et al., 2018, also reported that chlorine disinfection increases many extracellular antibiotic resistance genes, e.g., *erm*B, *tet*A, *tet*B, *tet*C, *sul*1, *sul*2, *sul*3, *amp*C, *aph*(2’)-Id, *kat*G and *van*A [66]. In particular, treated effluents from all months had contained *tetM* and *sul1* at the highest level, implying the widespread occurrence of these resistance genes in the effluent. The findings suggest that chlorination could attribute to the killing of ARBs and subsequent release of eARGs into effluents. The authors also proposed that *E.coli* was positively correlated with the measured eARGs. Consequently, this study concluded that human or animal-sourced fecal bacteria could be a significant source of eARGs in the effluents.

In a remarkable study, Mao and coworkers showed the abundance and diversity of eARGs in the river water and sediments [20]. The authors observed that a higher abundance of eDNA and eARGs (*sul1*, *sul2*,*tetW*, and *tetT* ) in sediments than in the water, implying the long persistence of eDNA in aquatic sediments. Therefore, it becomes clear that sediment-associated eDNA could be potential sources of ARGs in aquatic sediments.

The investigation by Zhang et al., 2013 was the first to report the presence of eARGs in environmental samples [24]. They investigated the eARGs levels in the sludge samples from cattle manure storage ponds and swine waste treatment lagoons. The detected antibiotic resistance gene were *sul*(I), *sul*(II), *tet*(O), *tet*(Q) and *tet*(X). Antibiotics, for example, chlortetracycline and tetracycline in the sludge, could promote the proliferation of eARGs by selective pressure. These findings substantially suggest that eDNA released into the environment become stable and widespread, which may contribute to the spreading of ARGs to allochthonous bacteria via natural transformation more than ever thought.

It is worth noting that the most critical antibiotic-resistant determinants, including carbapenem, were identified from eDNA that could lead to a potential public health threat. All these studies indicated that sediments could act as a reservoir for ARGs which can persist for an extended period. Furthermore, water samples in the study sites were found to be highly contaminated with sulfonamides (*sul1*, *sul2* and *sul3*). The relative abundance of eARGs in sediments was significantly higher than in the water samples. Therefore, it appears that depending on the status and abundance of the sediment-related bacterial community and eARGs, one can expect rippling effects on the proliferation of antibiotic resistance in aquatic systems. Additionally, these reviewed studies warrant that the relationships between community structure and eARGs will be investigated with respect to environmental variables, including physicochemical parameters, persistent contaminants (toxic metals) and antibiotics. This knowledge is crucial to understanding the sources and diversity of eARGs while under the clear influence of local anthropogenic activities.

## 5. Abiotic Factors Influence spread of ARGs in the Environment

Water is considered as an important channel and carrier of ARB and ARGs in the environment [67,68] and led to aquatic environments becoming persistent ARG reservoirs [9]. Many studies that have been conducted in aquatic environments suggest that abiotic factors, for example, pH [69], temperature [70,71], salinity [61,68,72], organic carbon [4], heavy metal contents on the co-selection of antibiotic resistance [73,74], antibiotic concentration [75,76], nutrients such as P and N [54], dissolved oxygen [54] and climatic factors such as rainfall [77] and seasonality [19] can affect the abundance of ARGs along with microbial communities in the environment. Indeed, the presence of various stressors like antibiotics and heavy metals may influence the eARGs transformation to susceptible bacteria in the environment by one of the natural-mechanism processes, so-called horizontal gene transformation (HGT). Therefore, the measurement of eARGs, together with stressors such as antibiotics and metal levels, and other abiotic factors, will provide comprehensive information regarding the prevalence and transmission risk in aquatic environments. Thus, eDNA present in waters could also be available for natural transformation of competent bacteria which might depend on various biotic and abiotic factors. On the other hand, the efficient binding of eDNA onto clay content of organic matter, humic substances and proteins cause higher accumulation and the persistence in sediments by reducing its decay rates and could contribute to horizontal gene transfer through natural transformation [12,78,79].

## 6. Treatment Technologies to Remove eDNA and eARGs

Until recently, a few studies had focused on technologies that will remove eARGs from the wastewater and are still in lab-scale practice. Cui et al., 2019, demonstrated that earthworm casts were found to be significant in removing the ARGs of both cell-associated and eDNA in the excess activated sludge [80]. Slipko et al., 2019, reported that membrane (ultrafiltration, nanofiltration and reverse osmosis) with a molecular weight cut off smaller than 5000 Da can remove eDNA effectively [81]. Furthermore, it was observed that membranes’ specifications, such as molecular weight cut-off and charge will be important parameters to remove eDNA from wastewaters. Interestingly, a report by Li et al. (2019) indicated that an integrated process of pre-coagulation and microfiltration was most useful to remove eARGs and dissolved organic carbon and phosphate [82]. They also found a significant reduction in membrane fouling. In another technology in the advanced treatment of the photo-Fenton process under visible LED light and neutral pH contributed to significant removal of eARGs, about 6.75–8.56 log as a result of shearing of eDNA [83]. However, a recent study emphasized that treatment by zero-valent iron or Fe^2+^ activated peroxydisulfate at an acidic pH resulted in the higher accumulation of eARGs in sewage sludge [65]. All these studies indicate that employing advanced and cost-effective technologies is critical to implementation in real wastewater plants to remove eDNA and its associated ARGs.

## 7. eDNA and Environmental Microbiomes

It has been reported that culture-dependent assessment of antibiotic resistance in environmental microbial communities will result in unrepresentative and biased results. Besides, there is convincing evidence which reports that less than 1% of microbes from environmental samples could be cultured [84,85]. An alternative is to use cultivation-independent methods such as metagenomics and NGS in combination. Such studies will provide comprehensive information regarding the microbial populations in communities and species richness and the ecological role in the environment [86]. It is important to understand biological processes and changes in the bacterial dynamics in the aquatic ecosystems for the assessment of human-driven changes and natural selection. Recently, several studies have demonstrated the dynamics of microbial populations by using eDNA deep sequencing in environmental matrices [23,57,87]. Gene-specific markers such as 16S ribosomal RNA (rRNA) gene deep sequencing could provide information about the different species of microbes present [57,86]. For instance, Yuan et al., 2019, investigated the difference in the composition of microbial communities using both iDNA and eDNA [57]. Although there was a shift in bacterial community composition, eDNA based sequencing revealed that proteobacteria were the most abundant phyla, and other phyla were reduced significantly as observed in iDNA sequencing. The authors proposed that eDNA originated mainly from proteobacteria by active and passive release into the environment. More recently, Oliveira et al. (2020) analyzed the shift in bacterial composition using the 16S rRNA V4 gene region in two different WWTP influents, effluents and reused effluents [23]. The bacterial community in the influents was dominated by Actinobacteria, Bacteroidetes, Firmicutes and Proteobacteria. Actinobacteria and Proteobacteria were dramatically increased in the effluents, suggesting the impact of the treatment processes. Although a significant difference observed in the relative abundance of microbial communities, the effluents and reused effluents’ bacterial communities have already presented in the influents. Thus, data on biological processes and microbiome could be used to develop the strategies to halt the antibiotic resistance.

## 8. Conclusion and Future Remarks

Although the last two years witnessed significant increase in the number of researches on the distribution of eDNA and its impact on environmental resistome, the source and fate in the environment largely remain overlooked. The impact of eDNA on the spread of ARGs is merely at its infancy level. Therefore, the extent to which eDNA contributes to HGT in allochthonous bacteria in the environment is still largely unknown and challenging. Despite this, ARGs encoded in the eDNA in aquatic sediments and water can potentially provide insights concerning the diversity of eARGs for the environmental antibiotic resistome. eDNA and its encoded eARGs released by the processes of cell death, lysis and secretion could persist in the environment matrices for long periods of time. Therefore, the acquisition of clinically significant eARGs (such as ESBL and carbapenem-resistant genes) can transform environmentally harmless bacteria into pathogens and may pose a potential threat to human health. Furthermore, the present review indicates the geographical distribution of studies only in a particular region. It may enable focusing on other areas globally to deeper understand the variation of climatic factors’ impacts and other environmental factors. CTAB based eDNA extraction method and the following qPCR as the most commonly used molecular approach used to understand the eARGs occurrence in aquatic environments. It should be noted that the CTAB methods currently used in the extraction of eDNA from environmental samples are based on the procedure developed by Corinaldesi et al., 2005 [79], and may be optimized for high yield with quality in future. It is to be noted that among the investigated countries, China was the country which had conducted most of the studies concerning the environmental occurrence of eARGs. Therefore, collectively, this review provides an insight into the contribution of eDNA to current environmental antibiotic resistome and may open a new perspective on the role of eDNA’s impact on the diversity of ARGs based on deeper sequencing studies. It is also noted that no investigations based from developing countries (Southeast Asia and Africa) where inadequate environmental regulations contributing to intensive anthropogenic contamination represents the key to research and filling the knowledge gap considering the coordinated global action for the mitigation of environmental antibiotic resistance. Future studies could also focus on the pristine environment as well as less or unaffected anthropogenic influenced environments to get more insights into eARGs. There are no studies to date for the transformation of environmental extracted eDNA in natural competent cells in the influence of various biotic and abiotic stresses in more realistic conditions mimicking occurrences in natural environments. Therefore, the study of the efficiency of environmental bacteria to take up eDNA via transformation will be of essential importance in future research which depends on advancements in methodological approaches. Overall, currently, novel and the most effective technology is needed to disinfect and remove ARBs and eARGs from wastewaters to control and limit the dissemination of antibiotic resistance and to maintain the ecological balance and decrease the risk to human health.

## Figures and Tables

**Table 1:** Commonly used eDNA isolation methods for water, sediment and sludge samples.

eDNA Isolation Method	Environment Sample Type	Filter Size and Type	Buffer Used	References
CTAB	river sediment	0.22 μm pore size, Polyvinylidene Fluoride (PVDF), Osmonics, U.S	NaH_2_PO_4_ (0.12 M, pH 8.0) and 0.2 g of polyvinyl polypyrrolidone (PVPP)	[20]
CTAB	activated sludge	0.02µm pore size, PVDF; Millipore, Bedford, MA, USA	NaH_2_PO_4_ (0.12 M, pH 8.0) and 0.1 g of PVPP	[50]
CTAB	estuarine sediments	0.02 µm membrane filter	NaH_2_PO_4_ (0.12 M, pH 8.0) and 0.2 g of PVPP	[61]
CTAB	coastal area: surface sea water and sediments	0.02 µm membrane filter	n.a	[62]
CTAB	sludge of livestock waste management structures	0.2 µm pore membrane	0.1 M phosphate buffer (PB, 0.093 M Na_2_HPO_4_ and 0.007 M NaH_2_PO_4_, pH = 8.0	[24]
CTAB	WWTP influents and effluents	0.02 µm membrane filter	n.a	[40]
CTAB	sediments of aquaculture farms	0.22 μm pore size, PVDF, Osmonics, U.S	NaH_2_PO_4_ buffer (0.12 M, pH = 8.0) containing 0.2 g PPVP	[21]
magnetic beads	water samples and activated sludge in WWTP	0.22 μm (Millipore, USA).	phosphate buffer (0.12M NaH_2_PO_4_, 0.12M Na_2_HPO_4_, pH = 4), 0.2g PVPP	[57]
NAAPs	water samples from river, lake and reservoir and drinking water	elute was filtered with polyethersulfone (PES) filter (0.45 μm, Millipore, USA).	eluent (15 g/L NaCl, 30 g/L tryptone, 15 g/L beef extract, 3.75 g/L Gly, 0.28 g/L Na(OH), pH = 9.3 ± 0.2)	[55]
NAAPs	tap water	elute was filtered using PES microporous membrane filter (0.45 μm, Millipore, USA)	n.a	[19]
hollow fiber ultrafiltration (HFUF) and silica binding	urban river water	polyestersulfone syringe filters with a pore size of 0.22 μm (Merck, Darmstadt, Germany)	sodium phosphate buffer (0.12 M Na_2_HPO_4_, 0.12 M NaH_2_PO_4_, 2% NaCl, pH = 8	[63]

n.a: not specified in the paper.

**Table 2:** eARGs detected quantitative real-time PCR (qPCR)-based methods in the eDNA isolated from environmental samples.

Antibiotic Class	Resistance Genes	Environments	Geographical Locations	References
Carbapenem Fluoro quinolones	*bla*_KPC_, *bla*_OXA-48_, *bla*_NDM_, *bla*_IMP_, *bla*_VIM_ *qnrA*, *qnrB* and *qnrS*	WWTP	Portugal	[23]
Sulfonamides Tetracycline β-lactams Chloramphenicol Erythromycin	*sul1*, *sul2*, *tetW*, *tetX*, *bla*_TEM_, *bla*_SHV_, *ampC*, *cat*, *cmr*, *ermA*, *ermB*,	Sludge sample WWTP, hospital, pharmaceutical industry, sediment samples from Aoyun lake and swine manure	China	[18]
Tetracycline, Sulfonamides, β-lactams	*TetC*, *tetM*, *sul1*, *sul2*, *bla*_TEM_, *qnrA* and *ampC*.	Tap water	China	[19]
Sulfonamides Tetracycline β-lactams	*sul1*, *sul2* *tetA*, *tetC*, *tetO* and *tetS* and *bla*_TEM-1_ and *bla*_nps-1_	Sediments from bullfrog farm and polyculture farm	China	[21]
tetracyclines, sulfonamides Fluoro quinolones	*sul1*, *sul2* *tetA, tetW* acc(6′)*-Ib,* qnr*S*	Biofilm, and sediment from estuary	China	[4]
tetracyclines, sulfonamides, β-lactams, macrolide	*TetA, tetC, tetM, tetX, sulI, sulII,blaTEM,ereA, ermB*	Wastewater treatment plant	China	[57]
tetracyclines, sulfonamides, macrolides, β-lactams	*tetC*, *sulII*, *ermB*, *Bla*_PSE-1_	WWTP	China	[40]
Sulfonamides Tetracyclines Macrolides β-lactams Quinolones	*sul1*, *tet(A)*, *ere(A)*, *bla*_TEM_, *qnrD*	Urban river water	Japan	[63]
tetracycline macrolide sulfonamide β-lactam	*tetM*, *tetW*, *tetG*, *tetX*, *ermB*, *ermF*, *mefA*, *ereA*, *sul1 sul2*, and *bla*_TEM_	Swine wastewater	China	[51]
sulfonamide, tetracycline	*sul(I)*, *sul(II)*, *tet(O)*, *tet(Q)* and *tet(X)*	Sludge of livestock waste management structures	USA	[24]
Aminoglycoside, tetracycline, chloramphenicol, sulphonamide, vancomycin, multidrug, beta-lactamase	*aac*(6′)-Ib−03, *aac*(6′)-II, *aadA*-02, *aadA*-01, *aadA*2 - 02, *aad*A2 - 01, *aad*A2 - 03, *strB*, *aad*A1, and *aad*A5 - 01), (*tetG*-01 and *tetM*-01), (*catB*3 and *floR*), (*catB*3 and *floR*), (*sul2*), (*VanC*-03), (*mexF*), (*bla*_VEB_)	Municipal sewage sludge	China	[65]
sulfonamides tetracyclines β-lactams fluoroquinolones	(*sul*1, *sul*2), (*tet*B and *tet*M), (*bla*_TEM_ and *bla*_OXA-1_), and (*qnr*S and *oqx*B).	Surface sea water from Coastal Bay	China	[62]
sulfonamides Tetracyclines	*sul*1- 3’ *CS*-TnAs3, *sul*2-*intI1*-IS*Vsa*3, and *tetX*-p63039	Sludge from WWTP	China	[50]
Macrolide, tetracycline, sulfonamide, β-lactam, aminoglycosides, rifampicin and vancomycin.	ermB, tetA, *tet*B and *tet*C, *sul*1, *sul*2 and *sul*3, *amp*C, *aph*(2’)-Id, *kat*G and *van*A.	Water sample from WWTP	China	[66]
sulfonamides Tetracyclines	*sul*1 and *sul*2 *tetW*, and *tetT*	Surface water and superficial sediment from river	China	[20]
ClassI integrons-integrase Aminoglycoside tetracycline, multidrug.	*int*I-1(clinic) *aadA01, aadA1,aadA-02,tetG*-*01, tetX, qacEdelta1*-*01, qacH*-*01*	Sediment samples from estuary	China	[61]
